# Cost of diseases related to alcohol consumption in the Brazilian Unified Health System

**DOI:** 10.1590/S1518-8787.2016050005741

**Published:** 2016-05-31

**Authors:** Evandro Silva Freire Coutinho, Luciana Bahia, Laura Augusta Barufaldi, Gabriela de Azevedo Abreu, Thainá Alves Malhão, Camila Ribeiro Pepe, Denizar Vianna Araujo

**Affiliations:** I Escola Nacional de Saúde Pública. Fundação Oswaldo Cruz. Rio de Janeiro, RJ, Brasil; II Departamento de Medicina Interna. Universidade do Estado do Rio de Janeiro. Rio de Janeiro, RJ, Brasil; III Departamento de Vigilância de Doenças e Agravos Não Transmissíveis e Promoção da Saúde. Secretaria de Vigilância em Saúde. Ministério da Saúde. Brasília, DF, Brasil; IV Instituto de Estudos em Saúde Coletiva. Universidade Federal do Rio de Janeiro. Rio de Janeiro, RJ, Brasil; V Unidade Técnica de Alimentação, Nutrição e Câncer. Coordenação de Prevenção e Vigilância. Instituto Nacional de Câncer José Alencar Gomes da Silva. Rio de Janeiro, RJ, Brasil; VI Medinsight Decisions in Health Care. São Paulo, SP, Brasil

**Keywords:** Alcohol-Related Disorders, economics, Health Care Costs, Costs and Cost Analysis, Unified Health System

## Abstract

**OBJECTIVE:**

To estimate the direct costs associated to outpatient and hospital care of diseases related to alcohol consumption in the Brazilian Unified Health System.

**METHODS:**

Attributable populational risks were estimated for the selected diseases related to the use of 25 g/day or more of ethanol (risk consumption), considering a relative risk (RR) ≥ 1.20. The RR estimates were obtained from three meta-analysis. The risk consumption rates of the Brazilian population ≥ 18 years old were obtained by a national survey. Data from the Hospital Information System of SUS (HIS-SUS) were used to estimate the annual costs of the health system with the diseases included in the analysis.

**RESULTS:**

The total estimated costs for a year regarding diseases related to risk consumption were U$8,262,762 (US$4,413,670 and US$3,849,092, for outpatient and hospital care, respectively).

**CONCLUSIONS:**

Risk consumption of alcohol is an important economic and health problem, impacting significantly the health system and society.

## RESUMO

**OBJETIVO:**

Estimar os custos diretos associados ao atendimento ambulatorial e hospitalar de doenças relacionadas com o consumo de álcool no Sistema Único de Saúde brasileiro.

**MÉTODOS:**

Riscos atribuíveis populacionais foram calculados para doenças selecionadas relacionadas ao uso de 25 g/dia ou mais de etanol (consumo de risco), considerando-se o risco relativo (RR) ≥ 1,.20. As estimativas de RR foram obtidas a partir de três meta-análises e as taxas de consumo de risco em brasileiros ≥ 18 anos obtidos em pesquisa nacional. Os dados do Sistema de Informações Hospitalares do SUS (SIH-SUS) e do Sistema de Informações Ambulatoriais do SUS (SIA-SUS) foram utilizados para estimar os custos anuais do SUS com as doenças incluídas na análise.

**RESULTADOS:**

Os custos totais estimados em um ano com todas as doenças relacionadas com consumo de risco foram US$8.262.762 (US$4.413.670 e US$3.849.092 para pacientes ambulatoriais e internados, respectivamente).

**CONCLUSÕES:**

Consumo de risco de álcool representa importante problema econômico e de saúde, com um impacto significativo para o sistema de saúde e para a sociedade.

## INTRODUCTION

Although alcohol consumption and related problems can vary around the world, the burden of alcohol-related diseases affects most countries. The World Health Organization (WHO, 2010)^a^ states that the use of alcohol is one of the main risk factors for poor health, premature deaths, disabilities, and global burden of disease, compromising both individual and social development. The WHO also estimates that, in 2004, 2.5 million individuals worldwide died of alcohol-related causes, which represents around 4.0% of the total number of deaths^a^. Thirteen percent of them were people aged between 15 and 29 years. Approximately 4.5% of the global burden of disease and injury is attributable to alcohol, although these figures can vary from 1.3% to 12.1% across the world^7^.

The cost of alcohol consumption to the health sector is not restricted to the treatment of alcohol dependence alone, but also to a set of alcohol-related diseases.^4^ Alcohol causes more than 30 diseases listed in the 10^th^ International Classification of Diseases (ICD-10), and works as a component cause for over 200 diseases^b^. The impact of alcohol use on these conditions depends on two aspects: the volume consumed and the pattern of drinking.

A systematic review^9^ reported that, despite the discrepancies in the estimation methods and cost components used across different studies, findings on the substantial economic burden of alcohol on society are consistent. The authors estimated that the economic burden of alcohol accounts for 0.5% to 5.4% of the gross domestic product. The socioeconomic development level of countries, regions, and individuals is inversely associated with the burden of alcohol-related diseases^c^. In order to estimate the global burden of harm related to alcohol, WHO classifies countries using a four-point risk scale (1 [low] to 4 [high]) according to the pattern of its alcohol consumption. Data have shown that this scale can be equated to morbid-mortality rates attributable to alcohol consumption. Brazil is classified as level 4^6^.

This study aimed to estimate the specific direct costs (medical costs) associated with outpatient and inpatient care of alcohol-related diseases in the Brazilian Unified Health System (SUS).

## METHODS

To assess health care costs for alcohol-related diseases, it is necessary to apply the population attributable risk (PAR) for alcohol to the number of patients with a related disease. The population attributable risk is the proportion of the incidence of a disease in the population exposed to a particular risk factor that would, consequently, be eliminated if exposure was eliminated.

For PAR calculation, we selected diseases based on two criteria: (i) relative risk (RR) for alcohol consumption ≥ 1.20, which included oropharyngeal cancer, esophageal cancer, laryngeal cancer, liver cancer, breast cancer, hypertension, liver cirrhosis, and chronic pancreatitis; and (ii) RR for alcohol consumption ≥ 1.10 and < 1.20, if the disease was considered a relevant public health problem because of its prevalence. No disease met this criterion.

The exposure category was 25 g/day or more of ethanol and the reference group was comprised of individuals referred to in the studies as abstemious.

We estimated PAR using Levin’s formula^5^, defined as the proportion of all cases that would not have occurred if the exposure had been absent.


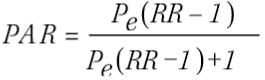


where *Pe* is the prevalence of exposure and *RR* the relative risk.

We searched for meta-analyses presenting RR estimates associated with the presence of alcohol consumption. As well as identifying the most recent meta-analyses, we also looked for large individual studies published after the search period covered by the meta-analyses. Literature was searched in Medline and Scopus databases. All the searches contained two blocks of concepts: one of the descriptors of exposure (“alcohol-related disorder*”, “alcohol drinking”, “alcohol intake”, “alcohol-related disease*”) and one of descriptors related to the selected diseases.

The meta-analyses quality was evaluated using the Assessing the Methodological Quality of Systematic Reviews (AMSTAR) inventory^8^. The meta-analyses carried out by Barnardi et al.^1^ (2001), Corrao et al.^2^ (2004), and Islami et al.^3^ (2010) provided the necessary information on RR for PAR calculation (Table 1).

**Table 1:** Characteristics and relative risks (RR) from the studies used in the analysis, by included diseases.

Disease	25 g/day RR	50 g/day RR	100 g/day RR	Study Meta-analysis*	Period covered	N and type of included studies; Total sample size	AMSTAR
Oropharyngeal cancer	1.86 1.73	3.11 2.77	6.45 5.75	Corrao et al.^2^ (2004) Bagnardi et al.^1^ (2001)	1966-1998 1966-2000	14 CC, 01 CO; 4507 25 CC, 01 CO; 7954	9/11 4/11
Esophageal cancer	1.39 T: 1.51 M: 1.43 F: 1.52	1.93 T: 2.21 M: 1.98 F: 2.24	3.59 T: 4.23 M: 3.49 F: 4.45	Corrao et al.^2^ (2004) Bagnardi et al.^1^ (2001)	1966-1998 1966-2000	13 CC, 01 CO; 3233 17 CC, 1 CO; 7239 17 CC, 01 CO; 3310 05 CC; 304	9/11 4/11
Laryngeal cancer	1.43 1.35	2.02 1.83	3.86 3.24	Corrao et al.^2^ (2004) Bagnardi et al.^1^ (2001)	1966-1998 1966-2000	20 CC; 3789 20 CC; 3759	9/11 4/11
Liver cancer	1.19 T: 1.20 M: 1.28 F: 1.97	1.40 T: 1.41 M: 1.51 F: 3.57	1.81 T: 1.83 M: 1.62 F: 9.15	Corrao et al.^2^ (2004) Bagnardi et al.^1^ (2001)	1966-1998 1966-2000	08 CC, 02 CO; 1321 16 CC, 03 CO; 1961 08 CC, 02 CO; 949 02 CC, 01 CO; 231	9/11 4/11
Breast cancer	1.25 1.31	1.55 1.67	2.41 2.71	Corrao et al.^2^ (2004) Bagnardi et al.^1^ (2001)	1966-1998 1966-2000	24 CC, 05 CO; 32175 37 CC, 12 CO; 44033	9/11 4/11
Hypertension	1.43	2.04	4.15	Corrao et al.^2^ (2004)	1966-1998	02 CO, 5801	9/11
Cirrhosis	2.90	7.13	26.52	Corrao et al.^2^ (2004)	1966-1998	06 CC, 03 CO; 2202	9/11
Chronic pancreatitis	1.34	1.78	3.19	Corrao et al.^2^ (2004)	1966-1998	02 CC, 247	9/11

T: total; M: male; F: female; CC: case-control study; CO: cohort study; AMSTAR: Assessing the Methodological Quality of Systematic Reviews

* Meta-analysis used as a reference for RR.

The prevalence rates of alcohol consumption in the adult population were obtained from a large national survey carried out by the Brazilian National Cancer Institute (INCA) in the 2002-2003 period^d^. The study defined one drink for women (15 g/day of ethanol) and two drinks for men (30 g/day of ethanol) as hazardous consumption. Thus, we used the RR provided in the meta-analyses of 25 g/day ethanol, as this was closest to the category that the INCA survey referred to as hazardous consumption. The assumption behind this decision was that the distribution of alcohol use over 25 g/day of ethanol is expected to be skewed to the right, which means that most individuals that consume more than 25 g/day are closer to this value than to the other extreme of the distribution.

The study report did not provide data stratified by sex, as its main objective was to estimate the prevalence of tobacco use. The figures by sex used in our analysis were kindly provided by INCA. Data were unavailable for nine of the 26 state capitals of Brazil. The median of the proportion of hazardous consumption from other capitals in the same region was used to replace the missing information.

Data on hospital admissions and outpatient visits were extracted from the Hospital Information System (SIH-SUS)^e^ and the Outpatient Information System (SIA-SUS)^f^. These open access databases provide aggregate information on the amount of money reimbursed by the government to the organizations that provide the health care (inpatient and outpatient) needed to treat and monitor these diseases.

We stratified the data by age (> 18 years), sex, type of service (inpatient or outpatient care), federated states, and year. Data were collected from 2008 to 2010 and the results reflect the average of the three years. This procedure was carried out to reduce the effect of random variation. Brazilian costs were converted into US dollars using a purchasing power parity basis: PPP 2010: US$1.00 = R$1.7^g^.

## RESULTS

The influence of alcohol consumption on the occurrence of the selected diseases, measured by PAR, showed a wide variation. The PAR estimates are presented in Tables 2 (male) and 3 (female). On average, the smallest PAR were for liver cancer and chronic pancreatitis among male adults, and breast cancer and chronic pancreatitis for female adults. Cirrhosis showed the highest PAR for both sexes.

**Table 2:** Population attributable risk (PAR) for selected diseases, by Brazilian capitals (male adult population).

Capital	Laryngeal cancer	Oropharyngeal cancer	Esophageal cancer	Liver cancer	Hypertension	Cirrhosis	Chronic pancreatitis
						
	PAR (%)	PAR (%)	PAR (%)	PAR (%)	PAR (%)	PAR (%)	PAR (%)
Porto Velho	4.98	8.75	4.17	3.03	4.58	17.48	3.65
Rio Branco	4.98	8.75	4.17	3.03	4.58	17.48	3.65
Manaus	5.74	10.03	4.81	3.50	5.28	19.76	4.22
Boa Vista	4.98	8.75	4.17	3.03	4.58	17.48	3.65
Belem	4.98	8.75	4.17	3.03	4.58	17.48	3.65
Macapa	4.98	8.75	4.17	3.03	4.58	17.48	3.65
Palmas	4.68	8.25	3.92	2.84	4.30	16.57	3.43
Sao Luis	7.71	13.26	6.48	4.74	7.10	25.24	5.70
Teresina	7.45	12.84	6.26	4.58	6.86	24.56	5.50
Fortaleza	7.96	13.66	6.69	4.90	7.33	25.89	5.88
Natal	7.10	12.27	5.97	4.36	6.54	23.61	5.24
Joao Pessoa	7.19	12.42	6.04	4.41	6.62	23.86	5.31
Recife	9.20	15.64	7.76	5.69	8.48	29.06	6.83
Maceio	7.45	12.84	6.26	4.58	6.86	24.56	5.50
Aracaju	5.61	9.80	4.70	3.42	5.16	19.37	4.12
Salvador	7.45	12.84	6.26	4.58	6.86	24.56	5.50
Belo Horizonte	6.78	11.74	5.69	4.15	6.24	22.72	5.00
Vitoria	7.53	12.97	6.33	4.63	6.94	24.77	5.56
Rio de Janeiro	6.05	10.54	5.07	3.69	5.56	20.65	4.45
Sao Paulo	3.68	6.53	3.07	2.22	3.37	13.37	2.69
Curitiba	3.74	6.63	3.12	2.26	3.43	13.57	2.73
Florianopolis	2.46	4.41	2.05	1.48	2.25	9.24	1.79
Porto Alegre	4.26	7.52	3.56	2.58	3.91	15.24	3.12
Campo Grande	4.24	7.50	3.55	2.57	3.90	15.19	3.11
Cuiaba	4.24	7.50	3.55	2.57	3.90	15.19	3.11
Goiania	4.24	7.50	3.55	2.57	3.90	15.19	3.11
Brasilia	6.02	10.49	5.04	3.67	5.53	20.56	4.43

**Table 3:** Population attributable risk (PAR) for selected diseases, by Brazilian capitals (female adult population).

Capital	Laryngeal cancer	Oropharyngeal cancer	Esophageal cancer	Breast cancer	Liver cancer	Hypertension	Cirrhosis	Chronic pancreatitis
							
	PAR (%)	PAR (%)	PAR (%)	PAR (%)	PAR (%)	PAR (%)	PAR (%)	PAR (%)
Porto Velho	1.47	2.66	1.23	0.66	2.99	1.35	5.70	1.07
Rio Branco	1.47	2.66	1.23	0.66	2.99	1.35	5.70	1.07
Manaus	1.47	2.66	1.23	0.66	2.99	1.35	5.70	1.07
Boa Vista	1.47	2.66	1.23	0.66	2.99	1.35	5.70	1.07
Belem	1.82	3.28	1.51	0.82	3.68	1.67	6.96	1.32
Macapa	1.47	2.66	1.23	0.66	2.99	1.35	5.70	1.07
Palmas	1.38	2.49	1.15	0.62	2.80	1.26	5.34	1.00
Sao Luis	4.02	7.11	3.35	1.83	7.95	3.69	14.46	2.94
Teresina	2.26	4.06	1.88	1.02	4.55	2.07	8.54	1.64
Fortaleza	2.44	4.38	2.04	1.11	4.92	2.24	9.20	1.78
Natal	2.16	3.88	1.80	0.98	4.36	1.98	8.20	1.57
Joao Pessoa	0.92	1.67	0.77	0.41	1.88	0.84	3.63	0.67
Recife	2.35	4.23	1.96	1.07	4.74	2.16	8.88	1.71
Maceio	2.26	4.06	1.88	1.02	4.55	2.07	8.54	1.64
Aracaju	1.99	3.58	1.66	0.90	4.02	1.82	7.59	1.45
Salvador	2.26	4.06	1.88	1.02	4.55	2.07	8.54	1.64
Belo Horizonte	3.32	5.91	2.77	1.51	6.61	3.04	12.18	2.42
Vitoria	3.66	6.50	3.06	1.67	7.28	3.36	13.32	2.68
Rio de Janeiro	2.84	5.09	2.37	1.29	5.70	2.61	10.58	2.07
Sao Paulo	1.11	2.01	0.92	0.50	2.26	1.01	4.33	0.80
Curitiba	0.80	1.46	0.67	0.36	1.64	0.73	3.16	0.58
Florianopolis	1.90	3.42	1.58	0.86	3.84	1.74	7.26	1.38
Porto Alegre	2.56	4.58	2.13	1.16	5.13	2.34	9.59	1.86
Campo Grande	2.08	3.73	1.73	0.94	4.19	1.90	7.89	1.51
Cuiaba	2.08	3.73	1.73	0.94	4.19	1.90	7.89	1.51
Goiania	2.08	3.73	1.73	0.94	4.19	1.90	7.89	1.51
Brasilia	1.75	3.15	1.45	0.79	3.54	1.60	6.70	1.27

Table 4 presents the total costs of the selected diseases, stratified by sex. Of the US$344 million spent on outpatient and inpatient care, 2.4% was attributable to alcohol consumption (≥ 25 g/day of ethanol). The total costs of these diseases were three times higher for women than for men. However, this pattern changed when the fraction attributable to alcohol consumption was considered, with men being responsible for more than 2/3 of the costs.

**Table 4:** Hospitalization, ambulatory, and total costs with estimated attributable costs of alcohol consumptiona related-diseases.

Hospitalization costs (US$)^b^	Hospitalization costs (US$) attributable to risk factors (%)	Ambulatory costs (US$)^b^ (medical visits, exams, procedures)	Ambulatory costs (US$) attributable to risk factors (%)	Total costs (US$)^b^ Outpatient and Inpatient care	Total costs (US$) attributable to risk factors (%)
Women		Women		Women	
41,6 million	571,3 thousand (1.37%)	218,2 million	1,7 million (0.82%)	259,8 million	2,3 million (0.91%)
Men		Men		Men	
42,7 million	3,2 million (7.67%)	41,5 million	2,6 million (6.33%)	84,2 million	5,9 million (7.01%)
Total		Total		Total	
84,3 million	3,8 million (4,56%)	259,7 million	4,4 million (1.70%)	344,1 million	8,2 million (2.40%)

^a^ 25 g/day or more of ethanol.

^b^ Three years average (2008-2010) of costs related to selected diseases (see Method’s section). Costs in Dollar PPP-2010.

When these costs were separately analyzed for in- and outpatient care, other patterns were observed. The total costs of hospitalization (Table 4) for the selected diseases were similar between men and women. However, the costs attributable to alcohol consumption were almost six times higher for the male group. For ambulatory services (Table 4), the total costs related to alcohol consumption were less similar between men and women than that observed for the hospitalization data. However, although women account for more than 80.0% of total costs for the selected diseases, this figure was reduced to 40.0% when the fraction attributable to alcohol consumption was considered.

The cost analysis by disease showed that oropharyngeal cancer, breast cancer, and cirrhosis were responsible for 34.0%, 20.0%, and 15.0% of the costs, respectively (Table 5). The first two diseases were also responsible for the larger proportion of costs in ambulatory services, while the relative impact of breast cancer on hospital costs was small. The most important diseases for hospital costs were cirrhosis and oropharyngeal cancer, followed by hypertension.

The costs attributed to alcohol in all selected diseases represented 0.38% of Brazilian gross domestic product.

**Table 5:** Attributable costs by alcohol consumptiona related-diseases in the Brazilian Unified Health System.

Disease	Hospitalization costs US$^b^	Ambulatory costs US$	Total US$
Breast cancer	136,1 thousand	1,4 million	1,6 million
Oropharyngeal cancer	1,1 million	1,6 million	2,7 million
Laryngeal cancer	277,5 thousand	367,4 thousand	645,0 thousand
Esophageal cancer	368,4 thousand	385,2 thousand	753,6 thousand
Liver cancer	70,8 thousand	21,9 thousand	92,8 thousand
Hypertension	630,8 thousand	443,4 thousand	1 million
Cirrhosis	1,2 million	10,0 thousand	1,2 million
Chronic pancreatitis	18,5 thousand	24,4 thousand	42,9 thousand
Total	3,8 million	4,4 million	8,2 million

^a^ 25 g/day or more of ethanol.

^b^ Three years average (2008-2010) of costs related to selected diseases (see Method’s section). Costs in Dollar PPP-2010.

## DISCUSSION

The estimates of the PAR showed a wide variation, but when data were analyzed both by capitals or sex, cirrhosis consistently had the largest PAR, followed by oropharyngeal cancer. Liver cancer showed a PAR similar to oropharyngeal cancer among women.

The average annual costs for the eight selected alcohol-related diseases were estimated at US$8 million. Treatment of men contributed significantly more to these costs, mainly because of the hospital sector. Thavorncharoensap et al.^10^ also reported a larger contribution of men to health care costs for alcohol related diseases in Thailand. Those authors observed that hospitalizations contributed slightly more than outpatient services, while in our study we observed the inverse. In Sweden, Jarl et al.^4^ found that around one-third of the health care costs in 2002 for the treatment of alcohol-related diseases derived from inpatients. The other two-thirds were equally shared by outpatients and primary care.

Comparing the health care costs found in our study with other studies is difficult due to several factors. In the systematic review carried out by Thavorncharoensap et al.^9^ (2009), studies were excluded for, among other reasons, not being published in English or if a full description of publication could not be retrieved. Their review found 20 studies that met their criteria, with 12 conducted in developed countries and only one in a developing country (Thailand). Their list of included diseases was larger than ours. For example, several studies included mental and behavioral disorders, injuries, poisoning, and external causes. Moreover, there were important differences in the economic and health care infrastructures, since nearly all these studies were carried out in developed countries.

Although in our study cirrhosis had the largest PAR for men and women, it reached the third position for attributable costs. Despite having a much smaller PAR than cirrhosis, oropharyngeal cancer occupied the first position for the costs impacting both hospital and ambulatory sectors. Breast cancer gave the second highest contribution for the costs, mainly because of the ambulatory sector.

Some limitations in our study need to be addressed, most of them resulting from the lack of information for the Brazilian population. First, PAR was estimated using RR estimated by meta-analyses with data from prospective and case-control studies carried out in high income countries. Our assumption is that the effect of alcohol consumption on the risk of the selected diseases is relatively homogeneous among western populations. We estimated PAR based on the RR for a consumption of 25 g/day of ethanol, since the Brazilian survey only provided information on the percentage of adults consuming one or more drinks for women (15 g/day of ethanol) and two or more drinks for men (30 g/day of ethanol). However, the probable impact of this decision is a small underestimation of the risk. Second, we had to assume that confounding factors were controlled when RR were estimated in order to interpret PAR as the proportion of cases that could be prevented if the exposure were eliminated. However, we have no information on how most of the individual studies included in the meta-analyses dealt with this methodological aspect. Third, we did not use exposure by age groups, since precise estimates were unavailable. Finally, the *Departamento de Informática do Sistema Único de Saúde* (DATASUS – Information Technology Department of the Brazilian Unified Health System) is an administrative database, designed to make and record payments for hospitalizations, rather than for epidemiological purposes. Many limitations regarding this data can be raised such as the quality of the input data, fraud, and duplication of data.

Another limitation concerns the findings of benefits related to moderate consumption of alcohol (e.g., coronary heart diseases) in some studies. Excluding these diseases from the analysis could have overestimated the costs.

The consumption pattern of alcohol in Brazil has been classified as being at a high-risk level^6^. To the best of our knowledge, this is the first study to estimate health care costs of relevant alcohol-related diseases in the SUS. This information is needed not only to improve the estimates of health care costs, but also to quantify the scale of the burden attributable to alcohol consumption on the health system and on society.

## Footnotes

a World Health Organization. Global strategy to reduce the harmful use of alcohol. Geneva; 2010. Available from: http://www.who.int/substance_abuse/msbalcstragegy.pdf

b World Health Organization. Global status report on alcohol and health. Geneva; 2011. Available from: http://www.who.int/substance_abuse/publications/global_alcohol_report/msbgsruprofiles.pdf

c World Health Organization. Global status report on alcohol and health. Geneva; 2011. Available from: http://www.who.int/substance_abuse/publications/global_alcohol_report/msbgsruprofiles.pdf

d Ministério da Saúde, Secretaria de Atenção à Saúde, Departamento de Regulação, Avaliação e Controle. Sistema de informações hospitalares do SUS – SIH/SUS. Brasília (DF): Datasus; 2011.

e Ministério da Saúde, Secretaria de Atenção à Saúde, Departamento de Regulação, Avaliação e Controle. Sistema de informações hospitalares do SUS – SIH/SUS. Brasília (DF): Datasus; 2011.

f Sistema de Informações Ambulatoriais do SUS - SIA. Suporte técnico ao Sistema de Informação Ambulatorial (SIA). Brasília (DF); s.d. [cited 2011 Aug 23]. Available from: http://sia.datasus.gov.br/principal/index.php

g World Bank Group. Washington (DC); s.d [cited 2012 May 1]. Available from: http://data.worldbank.org
